# Heterogeneous amplification of *ERBB2* in primary lesions is responsible for the discordant ERBB2 status of primary and metastatic lesions in gastric carcinoma

**DOI:** 10.1111/j.1365-2559.2011.04012.x

**Published:** 2011-11

**Authors:** Min A Kim, Hyuk-Joon Lee, Han-Kwang Yang, Yung-Jue Bang, Woo Ho Kim

**Affiliations:** 1 Department of Pathology, Seoul National University College of MedicineSeoul, Korea; 2 Cancer Research Institute, Seoul National University College of MedicineSeoul, Korea; 3 Departments of Surgery, Seoul National University College of MedicineSeoul, Korea; 4 Internal Medicine, Seoul National University College of MedicineSeoul, Korea

**Keywords:** fluorescence *in-situ* hybridization, HER2, immunohistochemistry, stomach neoplasm, tissue array analysis

## Abstract

**Aims:**

To determine the extent of *HER2* homogeneity/heterogeneity in primary versus metastatic gastric carcinoma (GC).

**Materials and results:**

The human epidermal growth factor receptor 2 (HER2) status in primary and metastatic lesions was evaluated by immunohistochemistry (IHC) and fluorescence *in-situ* hybridization (FISH). Four separate cohorts consisting of primary GC alone or primary GC paired with metastatic lesions were examined. In the FISH analysis of 325 primary GCs, eight cases (2.5%) showed amplification with a heterogeneous pattern, whereas 27 cases (8.3%) showed amplification with a homogeneous pattern, and in this cohort the discordant:concordant FISH ratio based on examination of three different areas in each primary lesion was 0.30:1. FISH testing using 250 paired primary and metastatic lesions revealed seven cases (2.8%) with discordant amplification. In metastatic disease positive conversion occurred in six cases (2.4%), whereas negative conversion happened in one case (0.4%). The discordant:concordant ratio of primary versus secondary lesions was 0.23:1. When the seven discordant cases were re-evaluated using whole sections of primary GCs, six showed a heterogeneous pattern of amplification.

**Conclusions:**

These findings suggest that the discordant *HER2* amplification observed in metastatic lesions is explained substantially by heterogeneity within primary tumours.

## Introduction

The *HER2* (*ERBB2*) gene encodes a 185 kDa transmembrane tyrosine kinase receptor (p185), which is a member of the epidermal growth factor receptor family.^1^,^2^ In breast carcinoma, *HER2* amplification is observed in 15–30% of cases and is known to be associated with adverse clinicopathological features and outcomes.^3^ In addition, human epidermal growth factor receptor 2 (HER2) is a powerful predictive marker of therapy based on the targeted HER2 inhibitor, trastuzumab.^4^–^7^ Conversely, in gastric carcinoma (GC), the frequency of *HER2* amplification has been reported variously to range from 7.7% to 27%, and this amplification has been established to correlate with an intestinal-type histology and poor survival.^7^–^11^ HER2-positive GC might also be a potential target for anti-HER2 therapy. Recently, the first randomized Phase III trial [Trastuzumab for GAstric cancer (ToGA)] showed that trastuzumab in combination with conventional chemotherapy is superior to conventional chemotherapy alone in HER2-positive advanced GC.^12^ Therefore, an accurate evaluation of HER2 status in GC has become increasingly important.

In breast cancer, *HER2* amplification and expression is highly homogeneous, although no consensus has been reached as to whether HER2 status should be assessed in primary or metastatic tissues for the selection of patients for anti-HER2 therapy in the metastatic setting. Although many studies have been conducted to resolve this issue, opinions differ. Some studies have shown good overall concordance between primary and metastatic lesions, but others have demonstrated high discordance rates.^13^–^19^

In GC, HER2 heterogeneity has not been researched extensively. In the literature, only one study has been performed using 49 pairs of primary and metastatic lymph node lesions, and the results obtained showed that in all cases primary and metastatic lesions were concordant by fluorescence *in-situ* hybridization (FISH).^20^ In our previous study, a few cases demonstrated heterogeneous staining in primary tumours, e.g. focal positivity for HER2 protein, and in these cases positive staining was observed in deeper portions or in foci of lymphatic invasion.^8^ In a separate experiment, discrepant HER2 staining results were obtained for 222 paired primary GCs and metastatic lesions in lymph nodes and in 3.6% of 222 cases, HER2 overexpression was observed in metastatic lesions only (positive conversion), and no case showed negative conversion.^21^ Although FISH was not performed, these findings suggest that some GCs either gain *HER2* copies during metastasis or that primary lesions have a heterogeneous HER2 status.

In order to examine further the heterogeneity of HER2 status between primary GC and metastatic lesions, 325 primary and 250 metastatic lesions were collected and evaluated by immunohistochemistry (IHC) and FISH.

## Materials and methods

### Patient Samples

Four cohorts of tissue samples resected or biopsied at Seoul National University Hospital between 1990 and 2006 were collected for analysis (Table 1). Cohort A comprised 325 cases of primary GC that were resected over 1 year (2004) – primary tumours of <3 cm were excluded and three different areas were examined per case; cohort B comprised 124 paired tissue samples of synchronous metastatic carcinoma to regional lymph nodes and primary GC tumours resected over 1 year (2004); cohort C comprised 65 paired tissue samples of synchronous distant metastasis and primary GC tumours; and cohort D comprised 61 paired tissue samples of metachronous distant metastasis and primary GC. Cohorts C and D were selected from archival tissues collected between 1990 and 2006 by reviewing medical and pathological records. Cases were enrolled if paired primary and metastatic tissues were available for IHC and FISH. Clinicopathological parameters, such as age, sex, histological type, pathological stage and interval of metastasis, were evaluated by reviewing medical charts and pathological records. This study was approved by the Institutional Review Board of Seoul National University Hospital (H-0809-066-257).

**Table 1:** Four cohort populations prepared for the study

Cohort	Number of cases	Examined sections	Description
A	325	975	Primary GC samples Examined three different areas per case

B	124	248	Paired samples Primary GC and synchronous metastatic carcinoma to regional lymph node

C	65	130	Paired samples Primary GC and synchronous metastatic carcinoma to distant site

D	61	122	Paired samples Primary GC and metachronous metastatic carcinoma to distant site

Total	575	1475	

GC, Gastric carcinoma.

### Slide Preparation for Evaluation of HER2

All tissue samples were fixed in 10% buffered formalin for 24–48 h and then embedded in paraffin. Representative cores (2 mm diameter) were taken from resected primary and metastatic lesions and 34 tissue microarray blocks were constructed using a trephine apparatus (Superbiochips Laboratories, Seoul, Korea). Non-neoplastic gastric mucosa specimens were included in each of the array blocks, and the tissue array blocks contained up to 60 cores. Small biopsy samples were evaluated using whole sections.

### Immunohistochemistry

Immunohistochemistry was performed using a Leica Bond-max automated immunostainer (Leica Microsystems, Newcastle, UK), as described by the manufacturer's protocol. In brief, formalin-fixed, paraffin-embedded, 4-μm sections were deparaffinized in a dry oven, dewaxed in xylene and rehydrated through graded alcohol. Heat pretreatment was performed using citrate buffer (pH 6.0) at 100°C for 20 min. Sections were then placed in an endogenous peroxide block for 5 min, and anti-HER2 antibody (A0485, rabbit polyclonal, 1:100; Dako, Carpenteria, CA, USA) was then applied for 30 min. Antibody binding was detected using a bond polymer refine kit (Leica Microsystems) and diaminobenzidine tetrahydrochloride solution (Kit HK153-5K; Biogenex, San Ramon, CA, USA) was used as a chromogen.

HER2 immunostaining was scored in accordance with the HER2 scoring system for GC (Hoffmann *et al.*’s criteria).^22^ This scoring system was applied to the tissue array and full section samples. Four grading systems were used: no membrane staining or membrane staining of <10% of tumour cells (score 0), faint/barely perceptible partial membrane staining in >10% of tumour cells (score 1+), weak to moderate staining of the entire or basolateral membrane staining in >10% of tumour cells (score 2+) and moderate to strong staining of the entire or basolateral membrane in >10% of tumour cells (score 3+). Biopsy samples with cohesive clones are considered positive irrespective of size. Scores of 0 and 1+ were considered negative and scores of 2+ and 3+ were considered positive (2 grading system).

### Fluorescence *In-Situ* Hybridization

Dual-colour PathVysion kits (Vysis, Downers Grove, IL, USA) were used for FISH. Briefly, slides of 2-μm-sectioned deparaffinized and dehydrated slides were incubated in 20% sodium bisulphate/×2 standard saline citrate (×2 SSC) at 43°C for 20 min. After being washed in ×2 SSC, slides were treated with proteinase K at 37°C for 25 min. Denaturation, hybridization and post-hybridization washing were carried out according to the manufacturer's instructions. Slides were then counterstained with 4′, 6-diamidine-2′-phenylindole dihydrochloride (DAPI) in anti-fade solution and examined under a fluorescence microscope (Olympus, Tokyo, Japan) equipped with Triple Bandpass Filter Sets (Vysis). After counting at least 20 tumour cell nuclei per slide, gene amplification was defined to be present when the FISH ratio, *HER2* signal (red)/centromere of chromosome 17 (green), was ≥2.0, as described by the manufacturer.

Cases showing heterogeneous amplification results of the three cores of primary tumour (cohort A) and primary and metastatic lesions (cohorts B, C and D) were re-evaluated using multiple whole sections of all available tumour tissues of paraffin blocks, including the used tissue array blocks and the remaining available blocks.

### Statistical Analysis

Differences were compared using Fisher's exact test or Pearson's χ^2^ test for non-continuous variables and using Student's *t*-test or analysis of variance for continuous variables. Survival curves were estimated using the Kaplan–Meier product–limit method and significant differences between survival curves were determined using the log-rank test. All statistical tests were two-sided, and statistical significance was accepted for *P*-values <0.05. All analyses were performed using SPSS version 17.0 (SPSS, Chicago, IL, USA).

## Results

### Comparison of HER2 Status as Evaluated by IHC and FISH in all Cohorts

A total of 1475 different tumour areas were evaluated for HER2 status (Table 1). Numbers of 0, 1+, 2+ and 3+ areas determined by IHC were 606 (41.1%), 474 (32.1%), 289 (19.6%) and 106 (7.2%), respectively (Table 2); thus, HER2 overexpression was observed in 395 areas (26.8%). Using FISH, 160 areas (10.8%) showed *HER2* amplification. None of the areas graded as 0 by IHC showed amplification by FISH, whereas all 3+ areas showed amplification. Three of the 474 areas (0.6%) graded 1+ by IHC were positive by FISH, whereas 51 of the 289 areas (17.6%) graded 2+ showed amplification. Furthermore, the correlation between IHC and FISH results was statistically significant (*P* < 0.001).

**Table 2:** Comparison of HER2 status between immunohistochemistry (IHC) and fluorescence *in-situ* hybridization (FISH) analysis in all different areas including cohorts A, B, C and D

	FISH		
	
	Not amplified	Amplified	Total
IHC			
0	606 (100.0%)	0 (0.0%)	606

1+	471 (99.4%)	3 (0.6%)	474

2+	238 (82.4%)	51 (17.6%)	289

3+	0 (0.0%)	106 (100.0%)	106

Total	1315	160	1475

*P* < 0.001 by Pearson's χ^2^ test.

**Figure 1:**
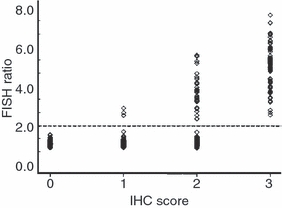
Scatterplot between ERBB2 immunohistochemistry (IHC) score and fluorescence *in-situ* hybridization (FISH) ratio in all 1475 different areas. The IHC score and FISH ratio correlated well (*P* < 0.001). All cases of IHC score 0 corresponded to FISH ratio <2.0, whereas all cases of IHC score 3+ were equal to or more than 2.0.

Figure 1 shows a scatterplot between IHC grades and FISH ratios in all 1475 different areas; IHC grades and FISH ratios were found to be well correlated (*P* < 0.001). For IHC grade 0, the FISH ratio was 1.09 ± 0.10, for grade 1+ the ratio 1.12 ± 0.20, for grade 2+ the ratio was 1.63 ± 1.08 and for grade 3+ the ratio was 4.99 ± 1.14.

### Primary Carcinomas Examined in Three Different Areas (Cohort A)

**Figure 2:**
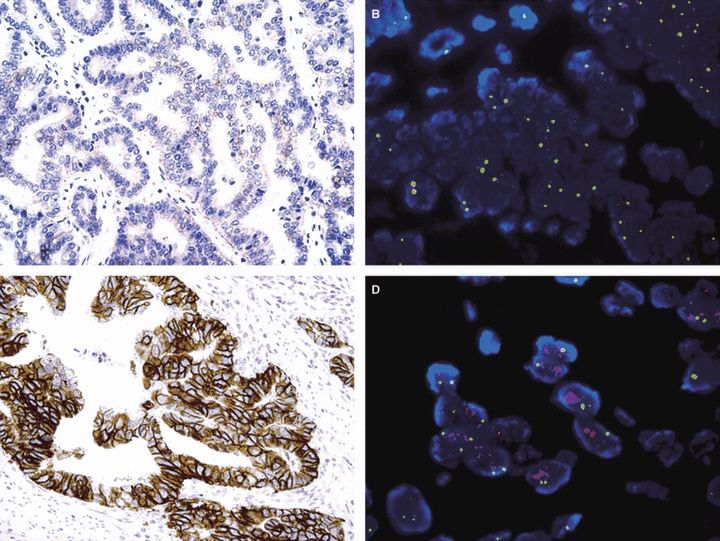
Heterogeneous ERBB2 status in different areas of the same primary tumour. ERBB2 protein expression evaluated by immunohistochemistry (IHC) (**A**,**C**) and gene amplification assessed by fluorescence *in-situ* hybridization (FISH) (**B**,**D**). **A**,**B**, 0 by IHC staining without amplification; **C**,**D**, 3+ by IHC staining with amplification.

Of the 325 cases examined, significant discordance in HER2 protein overexpression between the three different areas examined per sample was evident in 47 cases (14.5%) (Table 3). Using FISH, eight of the 325 cases (2.5%) showed discordant amplification in different areas and 27 cases (8.3%) showed concordant amplification in all three areas (Figure 2). Of the eight cases, seven cases demonstrated concordant IHC expression in three different areas, whereas one case demonstrated discordant IHC expression. Accordingly, the discordant:concordant ratio of FISH results in the three different areas of primary carcinoma was 0.30:1.

**Table 3:** Summary of HER2 immunohistochemistry (IHC) and fluorescence *in-situ* hybridization (FISH) in three different areas of primary gastric carcinomas (GCs) (cohort A)

	IHC overexpression	FISH amplification
None of three areas	220 (67.7%)	290 (89.2%)

One or two areas (discordant)	47 (14.5%)	8 (2.5%)

All three areas (concordant)	58 (17.8%)	27 (8.3%)

Total	325	325

Including homogeneously and heterogeneously amplified cases, 35 cases had *HER2* FISH-positive GC. Table 4 summarizes clinicopathological differences observed between primary GCs with or without *HER2* amplification. In particular, *HER2* amplified GCs were associated with the intestinal type of adenocarcinoma by the Lauren classification (*P* < 0.001). However, no differences were observed between *HER2* amplified and non-amplified cases in terms of age, sex or pathological stage. Kaplan–Meyer survival analysis also showed no difference between *HER2* amplified and non-amplified cases (data not shown).

**Table 4:** Comparison of clinicopathological findings between *HER2* amplified and non-amplified primary gastric carcinomas (GCs) evaluated by fluorescence *in-situ* hybridization (FISH) (cohort A)

	*HER2* FISH (*n* = 325)		
	
	None amplified in all three areas (%) (*n* = 290)	Amplified in at least one area (%) (*n* = 35)	*P*-value
Age (years)	58.2 ± 12.7	58.4 ± 11.9	NS

Sex			
Male	206 (71.0)	28 (80.0)	NS
	
Female	84 (29.0)	7 (20.0)	

Lauren classification			
Intestinal	128 (44.1)	26 (74.3)	<0.001
	
Diffuse	155 (53.4)	8 (22.9)	
	
Mixed	7 (2.4)	1 (2.9)	

Pathological stage			
I	124 (42.8)	17 (48.6)	NS
	
II	63 (21.7)	6 (17.1)	
	
III	55 (19.0)	3 (8.6)	
	
IV	48 (16.6)	9 (25.7)	

NS, Not significant.

### Paired Primary Carcinomas and Synchronous Regional Lymph Node Metastasis (Cohort B)

In total, 124 cases of paired primary GCs and synchronous metastatic lesions in regional lymph node tissues were included. Significant discordances were found between HER2 protein overexpression in primary and metastatic lesions in 27 cases (21.8%). Of these 27 cases, 19 were negative for HER2 overexpression in primary lesions but showed HER2 overexpression in metastatic lymph nodes (positive conversion), whereas eight cases showed overexpression in primary lesions and no overexpression in metastatic lesions (negative conversion).

**Figure 3:**
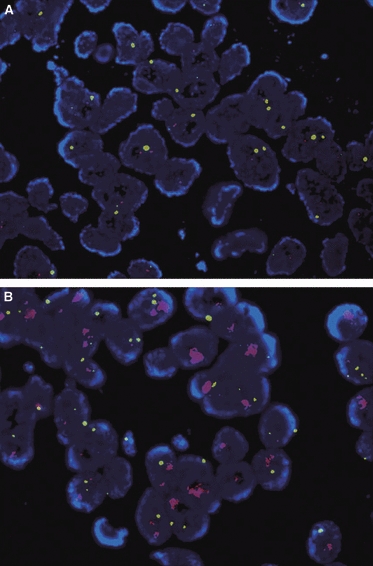
Discordant amplification result of *ERBB2* with fluorescence *in-situ* hybridization (FISH) analysis between primary and metastatic lymph node tissue. **A**, Not amplified in primary lesion. **B**, Amplified in metastatic lesion.

Five cases (4.0%) analysed by FISH showed discordance between primary and metastatic lesions (Table 5, Figure 3). Of the five cases, four cases demonstrated discordant IHC expression between the primary and the metastatic lesion, whereas one case demonstrated concordant IHC expression; four of these five cases also showed positive conversion and one case showed negative conversion. Twelve cases (9.7%) showed concordant amplification in primary and metastatic lesions. To confirm discordance, we performed IHC using multiple whole sections of primary and metastatic lesions. The representative area of each slide was then examined using multiple whole sections of primary and metastatic lesions using the FISH method. As a result, of the four that showed positive conversion, three cases showed heterogeneous amplification in both primary and metastatic lesions, and the remaining case was negative by FISH in the primary lesion and positive in metastatic lymph nodes. In the single case showing negative conversion the primary lesion was amplified heterogeneously, whereas the metastatic lesion was non-amplified homogeneously.

**Table 5:** Comparison of *HER2* fluorescence *in-situ* hybridization (FISH) result between primary gastric carcinomas (GCs) and metastatic carcinomas (cohorts B, C and D)

	Primary lesion		
	
	Non-amplified	Amplified	Total
Synchronous Lymph node Metastasis (cohort B)			
Non-amplified	107	1	108

Amplified	4	12	16

Total	111	13	124

Synchronous Distant Metastasis (cohort C)			
Non-amplified	53	0	53

Amplified	2	10	12

Total	55	10	65

Metachronous Distant Metastasis (cohort D)			
Non-amplified	52	0	52

Amplified	0	9	9

Total	52	9	61

Sum (cohort B, C, D)			
Non-amplified	212	1	213

Amplified	6	31	37

Total	218	32	250

### Paired Primary Carcinomas and Synchronous Distant Metastasis (Cohort C)

Sixty-five cases were included in this cohort. Metastasis occurred predominantly in liver (54 cases); other sites were the abdominal wall (four), supraclavicular lymph node (two), mesentery (two), diaphragm (one), rectal shelf (one) and uterus (one). Significant discordance was observed by IHC between primary and metastatic lesions in eight cases (12.3%). One case showed positive conversion and seven showed negative conversion.

Discrepant amplification was observed in two cases (3.1%), and both cases showed positive conversion (Table 5). Of the two cases, one case demonstrated discordant IHC expression between the primary and the metastatic lesion, whereas one case demonstrated concordant IHC expression. Ten cases (15.4%) showed concordant amplification in primary and metastatic lesions. For the confirmation of discordance, two discordant cases were re-evaluated using multiple whole sections of primary resected lesions using the IHC method. Representative areas of each slide were then examined using multiple whole sections of primary lesions using the FISH method. As a result, both cases showed heterogeneous amplification in the primary lesion. However, metastatic lesions could not be evaluated fully because tissue samples were small biopsy specimens.

### Paired Primary Carcinomas and Metachronous Distant Metastasis (Cohort D)

Sixty-one cases were included in this cohort. Metastatic sites were: liver (24 cases), abdominal wall (11), intestine (nine), uterus (five), supraclavicular lymph node (four), lung (two), breast (one) bone (one), skin (one), urinary bladder (one), adrenal gland (one) and gallbladder (one). The interval to proven metastasis ranged from 6 to 84 months, median 25.7 months. Using IHC, significant discordance was observed between primary and metastatic lesions in eight cases (13.1%). Seven cases showed positive conversion and one case negative conversion. Using FISH, nine cases (14.8%) showed amplification in both primary and metastatic lesions and no discordance was found between primary and metastatic lesions (Table 5).

### Summary of Metastatic Cases

In the present study, 250 metastatic lesions were compared with the corresponding primary GCs (cohorts B, C and D). IHC and FISH results were found to be well correlated in primary as well as secondary lesions (Table 6). In primary lesions, the amplification rate of cases with IHC grades 0, 1+, 2+ and 3+ was 0.0%, 2.8%, 17.6% and 100.0%, respectively, and in metastatic lesions the amplification rate of cases with IHC scores of 0, 1+, 2+ and 3+ was 0.0%, 1.4%, 17.5% and 100.0%, respectively. Overall, 43 cases showed a discordant IHC positivity result.

**Table 6:** HER2 immunohistochemistry (IHC) and fluorescence *in-situ* hybridization (FISH) results in 250 cases of paired primary and secondary gastric carcinomas (GCs) including cohorts B, C and D

	Amplification	
	
IHC	Primary lesion	Secondary lesion
0	0/106 (0.0%)	0/94 (0.0%)

1+	2/72 (2.8%)	1/73 (1.4%)

2+	9/51 (17.6%)	10/57 (17.5%)

3+	21/21 (100.0%)	26/26 (100.0%)

Total	32/250 (12.8%)	37/250 (14.8%)

When these 250 cases were examined using FISH, *HER2* amplification in primary GCs was detected in 32 (12.8%) and amplification in metastatic lesions in 37 (14.8%) cases (Table 7). Seven of the 250 cases (2.8%) showed discordant amplification between primary and secondary lesions and 31 (12.4%) cases showed concordant amplification, a discordant:concordant ratio of 0.23:1. Of these seven discordant cases, two cases showed a homogeneous IHC result, whereas five cases showed a different, heterogeneous, IHC result.

**Table 7:** Summary of *HER2* fluorescence *in-situ* hybridization (FISH) in primary gastric carcinomas (GCs) and metastatic carcinomas (cohorts B, C and D)

Cohort	B	C	D	Sum
Not amplified in any lesion	107	53	52	212 (84.8%)

Amplified in primary and/or secondary lesion	17	12	9	38 (15.2%)

Amplified in primary lesion	13	10	9	32 (12.8%)

Amplified in secondary lesion	16	12	9	37 (14.8%)

Concordant amplification	12	10	9	31 (12.4%)

Discordant amplification	5	2	0	7 (2.8%)

Positive conversion	4	2	0	6 (2.4%)

Negative conversion	1	0	0	1 (0.4%)

Total	124	65	61	250

The results of the re-evaluation conducted on whole sections of seven discordant cases are summarized in Table 8. Of the seven discordant cases, six showed heterogeneous amplification in the primary lesion and of these six, three were heterogeneous, two were positive and one was negative by FISH in the metastatic lesion. The remaining case was negative by FISH in the primary lesion, and positive in the metastatic lesion.

**Table 8:** Summary of discordant cases between primary and metastatic lesions by fluorescence *in-situ* hybridization (FISH)

	FISH in primary lesion			
	
	Homogeneously amplified	Heterogeneously amplified	Non-amplified	Total
FISH in metastatic lesion				
Homogeneously amplified	0	2*	0	2

Heterogeneously amplified	0	3	1	4

Non-amplified	0	1	0	1

Total	0	6	1	7

* Analysis of primary lesion was performed in the whole section from resected specimens and that of metastatic lesion in small biopsy specimens.

## Discussion

Heterogeneity of *HER2* gene amplification within a tumour and between primary and paired metastatic lesions is considered to be an important potential cause of treatment failure by molecular analysis-based targeted therapy in breast cancer. Some case reports have demonstrated the heterogeneity of *HER2* amplification in primary tumour, or discordant status between primary and metastatic lesions with regard to trastuzumab therapy in breast cancer patients.^23^,^24^ However, little is known of this topic in GC, although one IHC study showed that heterogeneity in GC is greater than that found in breast cancer.^22^ To clarify this issue, we collected 325 cases of primary GCs (cohort A) and 250 cases of metastatic carcinomas with their paired primary carcinomas (cohort B, C and D) and performed HER2 analysis by IHC and FISH.

Immunohistochemistry and FISH results in cohort A corresponded well. Almost all primary tumours with an IHC grade of 0 or 1+ showed no amplification by FISH, whereas all IHC 3+ cases showed amplification. Of the IHC 2+ cases, only about one-sixth showed *HER2* amplification. In this study, we used the HER2 scoring for GC established by Hoffmann *et al.*^22^ Our data relationship is similar to this consensus meeting. Also, the relationships are typical of those obtained in quality-controlled laboratories, and therefore the American Society of Clinical Oncology/College of American Pathologists Guidelines recommends that FISH analysis be conducted on cases with an IHC 2+ lesion in breast cancer.^25^ Furthermore, the concordance between IHC and FISH results found in primary gastric lesions was almost identical to that observed in the metastatic tumours.

Fluorescence *in-situ* hybridization analysis in cohort A showed that 35 cases (10.8%) demonstrated *HER2* gene amplification. Of these, 27 (8.3%) showed amplification with a homogeneous pattern, but eight (2.5%) showed heterogeneous amplification. Among the 325 cases of primary gastric carcinomas, 47 cases (14.5%) showed different IHC positivity, whereas 278 cases showed the same IHC positivity. Of the latter 278 cases, only one case (0.34%) demonstrated a different FISH result in three tissue array cores, while the remaining 277 cases showed the same FISH results in the three cores. In contrast, seven cases (14.9%) of the 47 former cases (heterogeneous IHC) demonstrated different FISH results in the three cores. As a result, we feel that one core or piece of biopsy is not sufficient for examination of positivity and/or amplification. Therefore, we believe that at least three different areas from the different paraffin blocks should be examined in order to overcome the heterogeneity in expression.

In addition, we compared the FISH results of primary and secondary lesions; the latter were composed of synchronous regional lymph node metastases (cohort B), synchronous distant metastases (cohort C) and metachronous distant metastases (cohort D). The FISH results of metachronous distant metastatic lesions agreed well with those of the primary lesions in all patients. However, *HER2* amplification in primary and secondary lesions was discordant in cohorts B and C in different ways; that is, from no amplification in primary lesions to positive amplification in secondary lesions, and vice versa. Overall for metastatic cases, the discordant:concordant ratio between primary and secondary lesions was 0.23:1, whereas the discordant:concordant ratio between different areas in primary tumours was 0.30:1. In other words, the discordance rate of primary versus metastatic lesions was no greater than the heterogeneous amplification rate within primary lesions. The incidence of discordant amplification for primary versus secondary lesions was 2.8% (seven of 250 cases), while the incidence of discordant amplification in different areas of primary lesions was 2.5% (eight of 325 cases). These findings suggest that discordant amplification in primary versus secondary lesions is the result of heterogeneity of amplification in primary lesions.

When we re-evaluated HER2 status using multiple whole sections (average five slides per cases) of the seven discordant cases, six showed heterogeneous amplification in the primary lesions. Only one case showed a negative FISH result in the primary lesion, but a positive FISH result in the metastatic lesion. According to our data, the IHC result of whole sections in the primary lesion and biopsy specimen were comparable in most of the heterogeneous cases (six of seven cases) expressed by FISH analysis. Therefore, we believe that assessment of metastatic disease is comparable to the primary lesion if the biopsy specimen is sufficient to overcome the heterogeneity of both lesions.

In metastatic lesions, positive conversion occurred in 2.4% and negative conversion in 0.4%, and although positive conversion was higher than negative conversion, the incidences of both were low. If it is presumed that a subclone with *HER2* amplification has a metastatic advantage, the positive conversion rate should have been much higher than observed and negative conversion should be non-existent. Accordingly, our findings indicate that *HER2* amplification promotes metastasis and recurrence only weakly. A similar result was observed in a study using breast cancer samples, in which of the 58 paired cases, positive conversion was found in 12% of patients and negative conversion in 2% only.^18^ However, conflicting results regarding discordant status in primary and metastatic lesions have also been reported in breast cancer. Gong *et al.*^16^ found 98% and 94% concordance between HER2 status in primary versus loco-regional recurrent lesions and in primary versus distant metastatic lesions, whereas Santinelli *et al.* evaluated HER2 status by IHC and FISH in primary and metastatic lesions and found relatively few cases with discordant status.^15^ Furthermore, in one GC study, no discrepant case was found by FISH between 49 paired primary and lymph node metastasis lesions.^20^ We believe that these controversial results were probably due to the small number of samples examined.

The present study was limited because we used a tissue array, not whole sections. However, in clinical practice IHC is usually performed on whole sections, while FISH analysis is applied to a small (2–3 mm) area. In this study, the IHC result was compared to the FISH result and it seems incongruous that while FISH was performed in a small area, the IHC study was performed on the whole section. For this reason we believe that using tissue arrays for the IHC study can result in reasonable comparison data between IHC and FISH. To overcome the inadequacy of the tissue array method, we prepared multiple tissue array blocks from three areas in every lesion. Furthermore, our tissue array slides consisted of 2-mm-diameter individual tumour tissue, which is a more than 10 times larger area compared to the conventional 0.6-mm core.

In summary, we found discordant *HER2* amplification in metastatic and primary lesions in 18.4% (seven of 38) and discordant amplification within primary tumours in 22.9% of cases (eight of 35). Thus, we believe that the discordant *HER2* amplification observed in metastatic lesions is explained largely by heterogeneity within the primary tumour. This study also shows that IHC correlate well with FISH results. In particular, none of the IHC grade 0 cases showed amplification whereas all IHC 3+ lesions showed amplification, and only about one-sixth of IHC 2+ cases showed *HER2* gene amplification. Therefore, we recommend that IHC 2+ cases should be analysed by FISH to determine *HER2* gene amplification in primary and metastatic lesions.

## Abbreviations

FISH: fluorescence *in-situ* hybridization
GC: gastric carcinoma
HER2: human epidermal growth factor receptor 2
IHC: immunohistochemistry
